# Analysis of 116 cases of rectal cancer treated by transanal local excision

**DOI:** 10.1186/1477-7819-12-202

**Published:** 2014-07-09

**Authors:** Gongping Sun, Yuanxin Tang, Xiaoxia Li, Jin Meng, Gaofeng Liang

**Affiliations:** 1 Department of Gastrointestinal Surgery, The Fourth Affiliated Hospital of the China Medical University, No. 4 Chongshan Road, Shenyang 110032, China

**Keywords:** rectal cancer, transanal local excision, survival, recurrence, radiotherapy

## Abstract

**Background:**

The purpose of this research was to evaluate the therapeutic effects and prognostic factors of transanal local excision (TAE) for rectal cancer.

**Methods:**

We retrospectively analyzed 116 cases that underwent TAE for rectal cancer from 1995 to 2008. A Cox regression analysis was used to analyze prognostic factors.

**Results:**

The survival times for the patients were from 14 to 160.5 months (median time, 58.5 months). The 5-year and 10-year overall survival rates were 72% and 53%, respectively. In all 16 cases experienced local recurrence (13.8%). Pathological type, recurrence or metastasis, and depth of infiltration (T stage) were the prognostic factors according to the univariate analysis, and the latter two were independent factors affecting patient prognosis. For patients with T1 stage who underwent adjuvant radiotherapy, there was no local recurrence; for those in T2 stage, the local recurrence rate was 14.6%. In addition, there was no difference between the patients who received radiotherapy and those who did not (T1: *P* = 0.260, T2: *P* = 0.262 for survival rate and T1: *P* = 0.480, T2: *P* = 0.560 for recurrence).

**Conclusions:**

The result of TAE for rectal cancer is satisfactory for T1 stage tumors, but it is not suitable for T2 stage tumors.

## Highlights

• Material for rectal cancer patients who underwent TAE were retrospectively collected.

• Survival rate and recurrence rate of the patients were analyzed.

• Recurrence or metastasis and depth of infiltration were prognostic factors.

• Adjuvant radiotherapy caused no significant clinical outcomes.

• Patients in T2 stage had a higher local recurrence rate.

## Background

Rectal cancer is the result of uncontrolled cell growth in the colon or rectum (parts of the large intestine), or in the appendix. It is a significant source of morbidity and mortality. The mortality rate in mainland China began to increase from 1995, especially in urban areas [1,2]. Low rectal cancer accounts for approximately 70% of the rectal cancers in China [3]. Surgical excision of the affected segment of the bowel is the mainstay treatment for rectal cancer [4].

Transanal local excision (TAE) is an acceptable curative operation for low rectal cancer [5]. This treatment at an early stage has multiple advantages, including non-invasion of the abdominal cavity, minimal trauma and no disturbance of urinary and sexual functions [6]. Meanwhile, TAE also makes it possible to avoid postoperative colostomy [7,8]. Currently, there are controversies about patient selection for TAE treatment. In this retrospective study, we investigated the efficacy of TAE treatment for rectal cancer patients at an early stage, as well as the prognostic factors in this treatment.

## Methods

### Patients

Clinical material for patients with rectal cancer treated between 1995 and 2008 by TAE at Liaoning Cancer Hospital and the Fourth Affiliated Hospital of the China Medical University were collected. The patients underwent a computed tomography (CT) scan, magnetic resonance imaging (MRI) scan and digital rectal examination. Patients within stage II of rectal cancer according to the National Comprehensive Cancer Network (NCCN) guideline were included, while those at a higher stage or who did not agree to the TAE regimen were excluded.

### Treatments

#### Surgical treatment

Preoperatively, evaluation of the tumors, including location, size, and stage, was performed using an enteroscope, pelvic CT/MRI and digital rectal examination [9]. Then, all tumors were treated by TAE. Dorsal lithotomy or the prone jackknife position was used according to the location of the tumor, that is, the distance from the anal verge to the distal tumor margin. Firstly, routine sterilization was conducted, followed by a 4- or 5-finger-wide expansion of the anus. Secondly, after groovy disinfection, the tumors were exposed. Finally, the tumors, together with the basal part and normal tissues within 1 cm of the lesions, were excised entirely. The excised specimens, resection margins and the basal parts were sent for pathological examination.

#### Adjuvant radiation therapy

After TAE surgery, 52 patients received adjuvant radiation therapy (RT) with the energy ranging from 6 to 10 MV according to their tolerance degree and the dose limitation for intestinal radiation. The detailed treatments including the radiation doses are presented in Table 1.

**Table 1:** Radiotherapy treatment for 52 patients

**Radioactive source**	**Doses (Gy)**	**Number of cases**
Cs-137	15–50	13
Ir-192	45	2
Cs-137 and Ir-192	15 + 30	2
Co-60	10–60	14
X-ray	21–67	21

#### Statistical analysis

Survival analysis was performed with the SPSS 15.0 package. The Cox proportional hazard model was used to analyze the prognostic factors. The univariate analysis used the Kaplan–Meier method, survival rates were assessed using a life table and comparisons between survival rates were performed using a log rank test (α = 0.05).

## Results

### Clinical material

A total of 116 cases (53 male and 63 female; 0.8:1.0) were included in this retrospective study. Their ages ranged from 30 to 80 with a median of 61. Preoperatively, 34 cases were diagnosed as T1, 77 cases as T2, and 5 cases as T3 cancers; and postoperatively, 7 cases were diagnosed as Tis, 24 cases as T1, 81 cases as T2, and 4 cases as T3. Besides, 52 patients received RT. All patients were treated with TAE, while one patient undertook a second TAE treatment. Six patients converted to a Miles operation, and one patient converted to a low anterior resection. Complications after the operation occurred in six cases (5.2%), among which five cases had a hemorrhage of more than 50 ml during the operation, and the bleeding was controlled after sufficient hemostatic measures and symptomatic treatment. One case developed a portal vein infection and septicemia, and the condition improved after anti-infective treatment.

### Follow-up and survival analysis

Two patients were lost to follow-up, while the follow-up rate was 98.3%. The survival time was 14 to 160.5 months, of which the median was 58.5 months. The 5-year and 10-year overall survival rates were 72% and 53%, respectively. In total, 16 patients developed a local recurrence of the tumor during the follow-up period (recurrence rate, 13.8%), while distant metastasis occurred in 13 cases (11.2%), and local recurrence combined with metastasis occurred in 3 cases (2.6%).

### Efficacy of adjuvant radiation therapy

**Figure 1:**
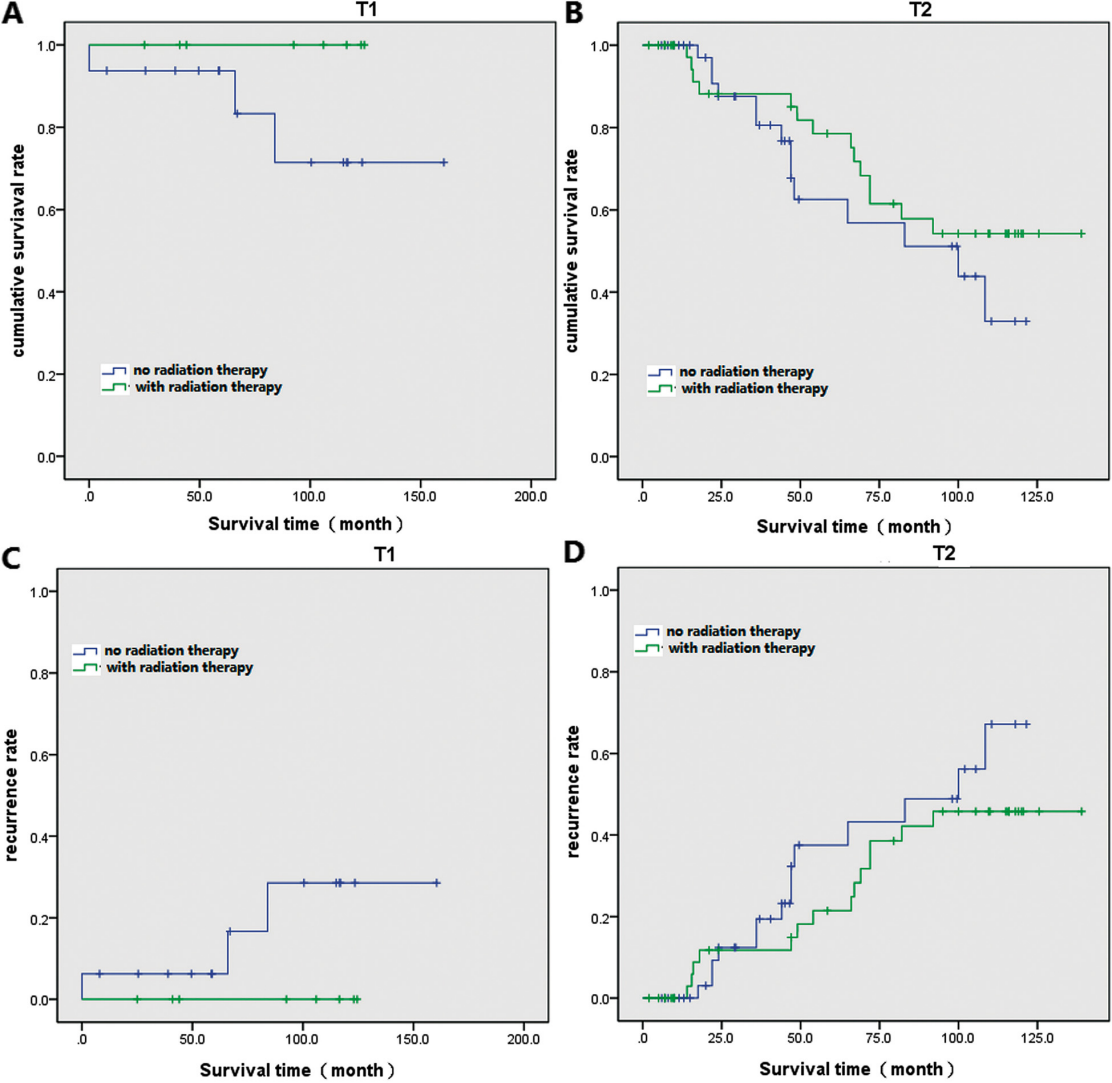
**Survival and recurrence rate for patients. (A)** Survival curve of patients at T1 stage. **(B)** Survival curve of patients at T2 stage. **(C)** Recurrence curve of patients at T1 stage. **(D)** Recurrence curve of patients at T2 stage.

Statistical comparisons of the survival rate and recurrence rate were performed between patients who received TAE and those received both TAE and adjuvant RT at the T1 and T2 stages (Table 2). No statistical difference in survival rate was found between the two groups (T1: *P* = 0.184 (Figure 1A); T2: *P* = 0.356 (Figure 1B)). In addition, there were also no significant differences in local recurrence rate between the TAE and TAE + RT groups at the T1 (*P* = 0.480, Figure 1C) and T2 stages (*P* = 0.560, Figure 1D).

**Table 2:** Survival and postoperative local recurrence for the T1 and T2 patients

**Stage**	**Therapy**	**Number of cases**	**Median survival time (months)**	**Survival rate (%)**		**Local recurrence (%)**	
				**5-year**	**10-year**	**5-year**	**10-year**
T1	TAE	16	67	75	38	6.3	6.3
	TAE + RT	8	99	63	50	0	0
T2	TAE	40	44	30	10	10.0	15.0
	TAE + RT	41	69	61	34	7.3	14.6

RT, radiation therapy; TAE, transanal local excision.

### Analysis of prognostic factors

We investigated the prognostic factors for TAE treatment using univariate analysis (Table 3). We found that pathological type, recurrence or metastasis, and depth of infiltration (T stage) were the prognostic factors. In the further Cox regression analysis, we detected that among the prognostic factors, recurrence or metastasis, and depth of infiltration were independent factors (Table 4) affecting patients’ prognosis. Patients with a higher T stage or with recurrence or metastasis have a higher risk of death.

**Table 3:** Univariate analysis of the prognostic factors

		**Survival rate (%)**			
**Clinical features**	**Cases (%)**	**5-year**	**10-year**	***P***	
Sex					
Male	53 (45.7)	64	58	0.912	
Female	63 (54.3)	77	49		
Radiotherapy		116		0.623	
Yes	52 (44.8)	75	55		
No	64 (55.2)	69	51		
Recurrence or metastasis	116				<0.01
Yes	32 (27.6)	32	—		
No	84 (72.4)	83	72		
Tumor diameter, *d* (cm)	116				0.928
*d* < 4 cm	109 (94.0)	71	54		
*d* ≥ 4 cm	7 (6.0)	75	—		
Distance to anal margin, *L* (cm)	115				0.128
*L* < 4 cm	37 (32.2)	87	58		
*L* ≥ 4 cm	78 (67.8)	67	51		
Proportion of tumor basal part, *C*	94				0.269
1/3 < *C*	69 (73.4)	69	52		
1/3 ≤ *C* < 2/3	24 (25.5)	70	46		
*C* ≥ 2/3	1 (1.1)	100	—		
Gross type	101				0.168
Protrusive	72 (71.3)	76	58		
Ulcerative	27 (26.7)	42	34		
Infiltrative	2 (2.0)	100	—		
Pathological type	115				0.025
Well differentiated	80 (69.6)	79	57		
Moderately differentiated	19 (16.5)	50	30		
Poorly differentiated	16 (13.9)	66	57		
Depth of infiltration	116				0.002
Tis	7 (6.0)	100	83		
T1	24 (20.7)	93	85		
T2	81 (69.8)	60	45		
T3	4 (3.4)	50	—		

**Table 4:** Multivariate Cox regression analysis of prognostic factors

**Clinical features**	***P***	**Exp (*B*)**	**95.0%confidence interval**
Sex	0.131	2.076	0.805–5.355
Age	0.186	1.022	0.990–1.056
Tumor diameter	0.434	1.270	0.698–2.312
Distance to anal margin	0.410	0.862	0.607–1.226
Width of tumor basal part	0.216	0.033	0.000–7.369
Gross type	0.610	1.251	0.529–2.956
Pathological type	0.305	1.304	0.785–2.165
T stage	0.042	2.622	1.037–6.625
Adjuvant radiotherapy	0.163	0.480	0.172–1.345
Recurrence or metastasis	<0.001	3.025	1.985–4.611

## Discussion

Reducing surgical complications and preserving the function of the anal sphincters are increasingly the focus of treatment of early local rectal cancer at stages T1 and T2. Local excision of rectal cancers, including TAE, transanal endoscopic resection and transsacral local resection, has been practiced for tens of years. Studies have constantly shown that for rectal cancer patients with tumors confined to the rectal wall, the outcomes of local excision are usually satisfactory [10,11]. Thus, local excision in the treatment of early rectal cancers is helpful and should be studied intensively.

Local excision of rectal cancers is an optional therapy for patients without lymph node metastasis, and its application depends on the depth of tumor infiltration, tumor differentiation and the extent of invasion to lymphatic and blood vessels [9,12]. According to treatment guidelines recommended by NCCN together with our clinical experience and most researchers’ viewpoints [13,14], we propose the indications for local excision should include: (1) a well-differentiated adenocarcinoma; (2) the depth of tumor invasion is at the Tis to T1 stage; (3) distal rectal cancer within 8 cm of the anal margin; (4) the diameter of the tumor is less than 3.0 cm and involves no more than 1/3 of the rectal perimeter; (5) no vascular infiltration; (6) no lymph node and distal metastasis; and (7) patient cannot tolerate major surgery or has developed severe complications.

Since lymph node dissection is impossible in a local excision, accurate preoperative staging is especially important in determining whether to use this method or not. The widely used staging measures include digital rectal examination, enteroscopy, endorectal ultrasonography, spiral CT, rectal MRI and positron emission tomography-CT [15-17]. We adopted CT, MRI and digital rectal examination.

The 5-year survival rates after a Miles resection, low anterior resection and ultra-low anterior resection are 70.3%, 72.9% and 73.7%, respectively, and for patients at stage I, the rate is 90.9% [7,9]. In our study, the overall 5-year survival rate was 72%, while the rates for T1 and T2 patients were 93% and 63%. This result demonstrates that for strictly selected cases, local excision is more suitable for T1 patients. In addition, the recurrence rate of T2 tumors in this study was far higher than that of T1 tumors, consistent with previous studies [18,19]. Therefore, we do not recommend the application of local excision for rectal cancer patients at stage T2.

Most relevant studies have reported that the reason for the high recurrence after local excision mainly lies in two aspects. One is the seeding implantation of cancer cells during the surgery. Though many clinicians recommend a washout during the operation, this does not necessarily lead to a reduction in the incidence of local recurrence [10]. In comparison, under the principles of no contact and whole resection, standard manipulations and appropriate case selection are more effective in preventing implantation of malignant cells. TAE is difficult to perform, and the field exposure is also very limited. The extent of the technical difficulty of a whole tumor excision is determined by multiple factors, including the tumor’s gross type, involved perimeter of rectal wall, distance from the anal margin, and patient’s age and obesity. In the NCCN guideline, one of the standards for TAE is a tumor diameter less than 3 cm, but there are also arguments that a standard of 2.5 cm might be more statistically significant [20]. For rectal cancers with low hazard factors, if the field is clear and manipulation is simple, then the tumor can be entirely excised while avoiding implantation of malignant cells.

The other chief factor responsible for the high recurrence after local excision is potential lymph node metastasis and insufficient excision of the marginal area. The probability of lymph node metastasis increases as T stage advances: at the T1 stage it is 0 to 12%, at the T2 stage it is 12 to 18%, and at the T3 and T4 stages it is up to 36 to 79% [10]. For rectal cancers involving the mucosa or submucosa, the probability of lymph node metastasis is 3% to 5%, thus local excision is a theoretically cure treatment. But for those with an infiltrated muscular layer, because the probability of lymph node metastasis is as high as 40%, it would be more prudent to consider adopting TAE to treat T2 rectal cancers. Our statistical analysis showed that as the T stage increases the incidence of postoperative recurrence tends to be higher, being 6.3% for T1 and 14.8% for T2, which is in accordance with other reports [8,21,22]. Therefore, local excision is only a palliative therapy.

This study shows that adjuvant radiotherapy can significantly decrease the recurrence hazards of T1 and T2 rectal cancer patients, but was not related to their clinical outcomes (T1: *P* = 0.184; T2: *P* = 0.356). The multimodality treatment schemas combining local excision and radiotherapy have been reported to be able to improve the local control rates, as well as better functional outcomes in patients with higher stage of rectal cancer [23,24]. Though no conclusion has been reached regarding the standards of the pre- and postoperative radiotherapy, and related background factors (such as the doses), most reports have indicated that compared with sole surgical treatment, surgery combined with preoperative radiotherapy can enhance the survival rate and lower the incidence of recurrence. Also, local excision was considered as an extended indication for T2-3 rectal cancer patients after neoadjuvant chemoradiotherapy [25]. Local excision is efficient in treating T2 patients with distal rectum cancer who exhibit complete pathologic response to preoperative chemoradiation [26]. However, in comparison, surgery with a postoperative radiotherapy will only decrease the tendency of recurrence while impose on effect on the survival rate [27,28]. Therefore, preoperative radiotherapy is strongly recommended for rectal patients.

## Conclusion

Our statistical analysis of the 116 TAE-treated rectal cancer cases indicates that TAE is an effective therapy for T1 cancers but it is not suitable for patients at the T2 stage. Tumor pathological type, recurrence or metastasis, and invasion depth (T staging) are all factors affecting patient prognosis. Tumors that are poorly differentiated and deeply infiltrated, and those with recurrence or metastasis, tend to have poor outcomes. Recurrence or metastasis and tumor infiltration depth are independent factors influencing survival conditions, and they could increase the hazard of death. Adjuvant radiotherapy could decrease the danger of local recurrence after surgery, but may not improve the survival rate. It can be considered a complementary and auxiliary therapeutic measure.

## Abbreviations

CT: computed tomography; MRI: magnetic resonance imaging; NCCN: National Comprehensive Cancer Network; TAE: transanal local excision.

## Competing interests

The authors declared that they have no competing interests.

## Authors’ contributions

GS and YT participated in the design of this study, and they both performed the statistical analysis. XL carried out the study, and, together with JM, collected important background information, and drafted the manuscript. GL conceived of this study, participated in the design and helped to draft the manuscript. All authors read and approved the final manuscript.
